# Tuning the Integration Rate of Ce(Ln)O_2_ Nanoclusters into Nanoparticulated ZrO_2_ Supports: When the Cation Size Matters

**DOI:** 10.3390/ma13122818

**Published:** 2020-06-23

**Authors:** Adrián Barroso-Bogeat, Iván Daza Raposo, Ginesa Blanco, José María Pintado

**Affiliations:** 1 Departamento de Ciencia de los Materiales e Ingeniería Metalúrgica y Química Inorgánica, Facultad de Ciencias, Universidad de Cádiz, Campus Río San Pedro s/n, 11510 Puerto Real (Cádiz), Spain; ivan.daza@iesdonana.org (I.D.R.); josemaria.pintado@uca.es (J.M.P.); 2 Instituto Universitario de Investigación en Microscopía Electrónica y Materiales (IMEYMAT), Facultad de Ciencias, Universidad de Cádiz, Campus Río San Pedro s/n, 11510 Puerto Real (Cádiz), Spain

**Keywords:** ceria-zirconia, rare-earth dopants, thermochemical ageing treatments, redox behaviour, X-ray photoelectron spectroscopy

## Abstract

Three nanostructured catalysts with low total rare earth elements (REEs) content (i.e., 15 mol.%) were prepared by depositing CeO_2_ or Ln^3+^-doped CeO_2_ (Ln^3+^ = Y^3+^ or La^3+^; Ln/Ce = 0.15) on the surface of ZrO_2_ nanoparticles, as nanometre-thick, fluorite-type clusters. These samples were subjected to successive reduction treatments at increasing temperatures, from 500 to 900 °C. A characterisation study by XPS was performed to clarify the diffusion process of cerium into the bulk of ZrO_2_ crystallites upon reduction to yield Ce_x_Zr_1−x_O_2−δ_ surface phases, and the influence of the incorporation of non-reducible trivalent REE cations, with sizes smaller (Y^3+^) and larger (La^3+^) than Ce^4+^ and Ce^3+^. For all nanocatalysts, a reduction treatment at a minimum temperature of 900 °C was required to accomplish a significant cerium diffusion. Notwithstanding, the size of the dopant noticeably affected the extent of this diffusion process. As compared to the undoped ZrO_2_-CeO_2_ sample, Y^3+^ incorporation slightly hindered the cerium diffusion, while the opposite effect was found for the La^3+^-doped nanocatalyst. Furthermore, such differences in cerium diffusion led to changes in the surface and nanostructural features of the oxides, which were tentatively correlated with the redox response of the thermally aged samples.

## 1. Introduction

Since the middle of the 1990s, cerium-zirconium mixed oxides (Ce_x_Zr_1−x_O_2_) have been extensively employed in the field of heterogeneous catalysis either, as catalysts on their own, or much more frequently, as supports of a variety of metal-containing catalytic active phases [1,2]. Such an extensive use is chiefly ascribable to their improved thermal stability, surface area, and redox properties (i.e., reducibility and the coupled oxygen storage capacity, OSC), as compared to pure ceria (CeO_2_), another oxide commonly found in the formulations of catalysts [3,4,5]. In fact, because of these advantages, cerium-zirconium mixed oxides successfully replaced pure ceria as the oxygen buffer component in the latest generations of three-way catalysts (TWC) in internal combustion engine vehicles, for the removal of post-combustion pollutants [6,7,8]. Additionally, the aforementioned enhanced properties of these mixed oxides may be further modified in a controlled way, by incorporating small amounts of other rare-earth elements (REEs, henceforward) [3,9,10], on the one hand, or by applying different thermochemical ageing treatments under both oxidizing and reducing atmospheres, on the other [8].

A number of studies have revealed that the incorporation of non-reducible trivalent REE cations (referred to as Ln^3+^ hereafter), such as Y^3+^ and La^3+^, into the lattice of Ce_x_Zr_1−x_O_2_ solid solutions, may lead to significant modifications, affecting not only their crystalline structure, but also their thermal stability, oxygen mobility, redox properties, and OSC [9,10,11,12,13,14,15,16,17,18,19,20,21,22]. Most of this literature is essentially devoted to analysing, in detail, the influence of doping on the aforesaid properties for bulk materials, i.e., when zirconium, cerium, and the dopant are completely integrated, forming single-phase solid solutions. Nevertheless, to the best of our knowledge, there are no reports concerning the effects of the dopant when the formation of the Ln^3+^-doped Ce_x_Zr_1−x_O_2_ is constrained to the outermost surface layers of nanoparticulated ZrO_2_ supports.

As far as the application of thermochemical ageing treatments is concerned, it has been generally agreed that the temperature of the re-oxidation treatment applied to heavily reduced Ce_x_Zr_1−x_O_2_ solid solutions may reversibly modify their redox behaviour [8,23,24,25,26,27,28,29,30]. Thus, as previously reported by Baker et al. [26], the application of successive redox cycles consisting of reduction in a flow of H_2_ (5%)/Ar at 950 °C, followed by alternate re-oxidation treatments in a flow of O_2_ (5%)/He at mild (550 °C) and high (950 °C) temperature, results in oxide samples exhibiting markedly different, but reversible, redox behaviours. The mild temperature re-oxidation leads to enhanced low-temperature reducibility, whereas the opposite applies to the high temperature re-oxidation, i.e., the severe deterioration of low-temperature reducibility. Furthermore, it should be also highlighted that the above-described chemical effects are accompanied by and closely related to relevant modifications in the mixed oxide crystalline structure [24,28,31,32,33,34,35,36]. By using a set of structural characterization techniques, including X-ray diffraction [24,28,31], Raman spectroscopy [24,28,33,37], electron microscopy [5,32,34,38,39], and fluorescence line-narrowing spectroscopy [30], a number of works have evidenced that the reduction at high temperature of a fresh Ce_x_Zr_1−x_O_2_ solid solution with a completely random distribution of Ce^4+^ and Zr^4+^ cations promotes a certain ordering in the cationic sublattice, ultimately leading to a pyrochlore-type structure (Ce_2_Zr_2_O_7_). Further re-oxidation of this heavily reduced mixed oxide sample at moderate temperature (i.e., below 600 °C) does not induce any modification in the ordered cationic sublattice of pyrochlore [8,24,35]. In stark contrast, its re-oxidation at high temperature (i.e., above 900 °C) results in a strong rearrangement of the cationic sublattice back to the starting random distribution [8,24,28].

On the other hand, the growing technological applications of ceria and other REE oxides, together with the relative scarcity and limited geographical location of their main economically viable ores, have led to a sharp rise in prices since 2011 [40,41,42]. Because of this situation, reducing and optimizing the total REEs content in the formulations of ceria-zirconia-based catalysts, while preserving, or even improving, their excellent redox properties and catalytic performances, have become topics currently receiving a great deal of attention and encouraging research efforts [38,39,42]. Bearing in mind that heterogeneous catalysis is considered to be an essentially surface phenomenon, a feasible strategy to fulfil the aforementioned challenging goal could rely on depositing ceria or Ln^3+^-doped ceria as epitaxially grown nanometer-thick layers on the surface of highly crystalline nanoparticulated ZrO_2_ supports. The formation of the desired surface ceria-zirconia phase with pyrochlore-related structure would be accomplished in a subsequent preparation step, by simply subjecting the fresh sample to successive thermochemical ageing treatments under reducing and oxidizing atmosphere at appropriate temperatures.

Within this context, the present work is firstly intended to develop a novel nanostructured material, showing potential application in actual catalytic systems, by coating commercial ZrO_2_ nanoparticles with nanometer-thick ceria clusters, by using a facile surfactant-free wet chemistry route. Secondly, the integration process between both oxides as a result of the application of a series of thermochemical ageing treatments under reducing and oxidizing conditions, and leading to the formation of a surface mixed oxide phase is systematically studied in detail for the first time. Finally, the influence on the integration process of the incorporation in the surface ceria phase of small amounts of Ln^3+^ dopants is also analysed and correlated with the redox response of the thermally aged nanomaterials. For such an aim, a couple of Ln^3+^ cations exhibiting markedly different radii, as compared to that of Ce^4+^ and Ce^3+^ (i.e., *r* = 111 pm and 128 pm, respectively [43]), have been selected [44]: one with smaller size (Y^3+^, *r* = 102 pm [45,46]), and another having larger size (La^3+^, *r* = 132 pm [43]). The obtained results are expected to be rather interesting and useful, with a view to designing novel nanostructured catalysts, with low lanthanide content and improved properties, by simply tuning the diffusion of the CeO_2_-based surface phase on the ZrO_2_ support.

## 2. Materials and Methods

### 2.1. Synthesis of ZrO_2_-CeO_2_ and ZrO_2_-Ce_0.87_Ln_0.13_O_1.935_ Nanostructured Materials

As received without any further treatment, non-porous commercial ZrO_2_ nanoparticles purchased from SkySpring Nanomaterials (Houston, TX, USA), roughly spherical with 20 to 30 nm average particle size, were employed as support. CeO_2_ nanoclusters were deposited on ZrO_2_ surface by a surfactant-free controlled chemical precipitation method, using Ce(NO_3_)_3_ as precursor and hexamethylenetetramine (HMT, hereafter) as precipitant agent, in accordance to the procedure previously described in detail elsewhere, for the synthesis of both SiO_2_-CeO_2_ and ZrO_2_-CeO_2_ core@shell nanocomposites [42,47,48]. Briefly, 1 g of ZrO_2_ nanoparticles and the amount of Ce(NO_3_)_3_·6H_2_O (99.99%, Sigma Aldrich, Steinheim, Germany) required for attaining a 15% CeO_2_ molar loading in the final product were thoroughly dispersed in 60 mL of ethanol (96% vol., VWR Chemicals, Briare, France), under ultrasonication for 30 min. The resulting suspension was heated from room temperature up to 75 °C, under continuous magnetic stirring of 800 rpm. Then, a solution prepared by dissolving the appropriate amount of HMT (i.e., HMT to Ce^3+^ molar ratio of around 5) in 20 mL of deionized water was slowly added to the above suspension at a rate of 0.33 mL·min^−1^, by using a syringe pump specially designed for dosing small fluid volumes with high precision. The resulting reaction mixture was further aged for 1 h under the aforesaid heating and stirring conditions. Once this time had elapsed, the obtained solid was separated by centrifugation, repeatedly washed with deionized water until neutral pH to remove any possible remnants of reagents, and then with ethanol, and oven-dried at 80 °C overnight. Finally, the oven-dried product, in a series of successive steps, was ground, sieved, and calcined at 350 °C for 2 h in air, to obtain the ZrO_2_-CeO_2_ nanocomposite. Considering the specific surface area value of around 35 m^2^·g^−1^ for the ZrO_2_ nanoparticles employed as a support, the nominal loading of 15 mol.% is approximately equivalent to a coverage by three CeO_2_ monolayers.

Following an identical synthesis procedure, two additional nanocomposite samples were also prepared in parallel, by incorporating Ln^3+^ cations (i.e., Y^3+^ or La^3+^) in the supported CeO_2_ nanoclusters, with a Ln to Ce atomic ratio of 15%. The Ce_0.87_Ln_0.13_O_1.935_ mixed oxide nominal molar loading in the resulting materials was set again at 15%, in order to allow a proper comparison between undoped and doped samples. Similar to the preparation of the ZrO_2_-CeO_2_ nanocomposite, the employed precursors were Y(NO_3_)_3_·4H_2_O and La(NO_3_)_3_·6H_2_O, both being of 99.99% purity and supplied by Sigma Aldrich (Steinheim, Germany). The as-prepared ZrO_2_-CeO_2_ and ZrO_2_-Ce_0.87_Ln_0.13_O_1.935_ nanostructured materials will be hereinafter referred to as “fresh samples”.

### 2.2. Thermochemical Ageing Treatments

**Figure 1:**
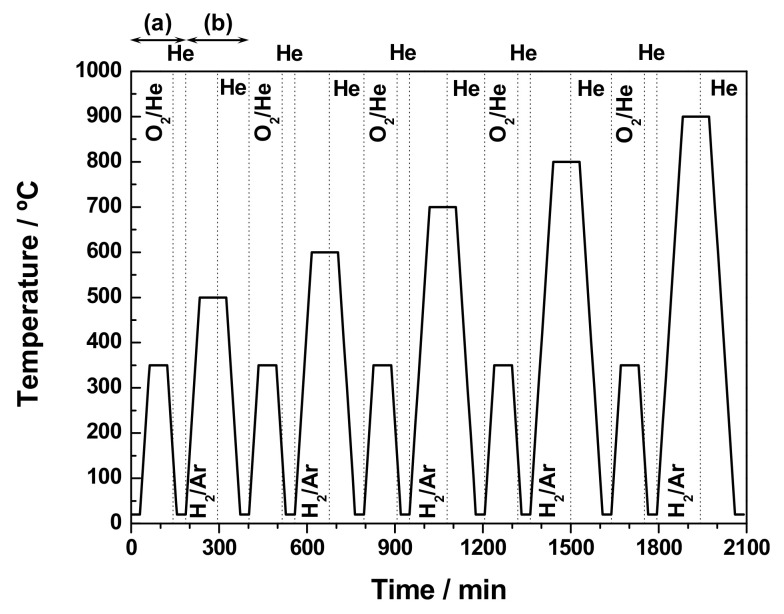
Schematic description of the stages involved in the successive thermochemical ageing treatments applied to the fresh undoped and Ln-doped nanostructured ZrO_2_-CeO_2_ samples. The heating rate was 10 °C·min^−1^ in all cases.

In order to gain a better insight into the integration process of surface CeO_2_ nanoclusters into the nanoparticulated ZrO_2_ substrate, as well as the influence of the incorporation of Ln^3+^ cations with varying sizes, portions of the fresh samples were subjected to successive thermochemical ageing treatments under alternate oxidizing and reducing conditions. Such treatments are described in detail below and schematized in Figure 1.

The fresh sample (i.e., either ZrO_2_-CeO_2_ or ZrO_2_-Ce_0.87_Ln_0.13_O_1.935_), after a mild oxidation treatment consisting of heating in a 60 cm^3^·min^−1^ STP flow of O_2_ (5%)/He from room temperature up to 350 °C at a rate of 10 °C·min^−1^ followed by a holding time of 1 h at such temperature, was slowly cooled down under the same oxidizing gas mixture to 150 °C, and then the flow was switched to He for further cooling down to room temperature (steps labelled as (a) in Figure 1). Subsequently, this first redox ageing cycle was concluded by applying a reduction treatment performed in a flow of 60 cm^3^·min^−1^ STP of H_2_ (5%)/Ar from room temperature, up to 500 °C at a heating rate of 10 °C·min^−1^. The sample was held at this maximum temperature for 1 h. Once such period of time had elapsed, the flow was then changed to He and after 30 min at the above temperature the sample was allowed to slowly cool down to room temperature under this inert atmosphere (stages referred to as (b) in Figure 1). Successive redox ageing cycles (i.e., steps (a) and (b)) were carried out on the resulting reduced sample following the above-described pattern of consecutive oxidation-reduction treatments. Nonetheless, while the re-oxidation temperature was set at 350 °C, the reduction temperature was increased stepwise by 100 °C in each of the successive cycles, up to a maximum of 900 °C. At this point, it should be highlighted that, immediately after each reduction treatment, the resulting samples were submitted to a careful passivation protocol, in order to avoid, as far as possible, their fast and uncontrolled re-oxidation by sudden contact with air during their transport, storage, and subsequent physico-chemical characterization. Such a protocol consisted of cooling down the reduced materials at −90 °C under a 60 cm^3^·min^−1^ STP flow of pure He, switching to O_2_ (5%)/He at the same temperature, and then slowly warming up to room temperature [49]. The thermally aged samples resulting from each of these oxidation-reduction experiments will be henceforth referred to as “XXX”, where XXX stands for the maximum temperature of the last applied reduction treatment. Accordingly, “ZrO_2_-CeO_2_-700” denotes the sample resulting from the application of consecutive reduction treatments at 500, 600, and 700 °C, after a re-oxidation step at 350 °C in each case.

Furthermore, in order to rule out any possible modifications ascribable to thermal sintering and phase transition of the support and affecting the conclusions about the integration process, an aliquot of the pristine ZrO_2_ nanoparticles was subjected to the previously described set of consecutive thermochemical ageing treatments.

### 2.3. Characterization of the Fresh and Thermally Aged ZrO_2_-CeO_2_ and ZrO_2_-Ce_0.87_Ln_0.13_O_1.935_ Samples

#### 2.3.1. Chemical Composition

The actual CeO_2_ and Ce_0.87_Ln_0.13_O_1.935_ loading of the fresh nanocomposites, as well as the corresponding Ce/Zr, (Ce + Ln)/Zr, and Ln/Ce atomic ratios, were estimated by X-ray fluorescence (XRF) analyses, performed using a M4 Tornado energy dispersive spectrometer from Bruker (Billerica, MA, USA), equipped with Mo K*α* radiation (*λ* = 0.7107 Å) source, operating at 50 kV and 600 μA.

#### 2.3.2. X-ray Diffraction

Powder X-ray diffraction (XRD) patterns for both the fresh and thermally aged samples were recorded at room temperature on a D8 ADVANCE diffractometer from Bruker (Billerica, MA, USA), with Cu K*α* radiation (*λ* = 1.5406 Å), under the following acquisition conditions: 2*θ* range from 10° to 75°, step size 0.02°, and step counting time 38.4 s. The software DiffracPlus (Bruker, Karlsruhe, Germany) was employed, to determine the full width at half maximum (FWHM) of the (111) diffraction peak for monoclinic ZrO_2_ and the (111), for both supported fluorite-type CeO_2_ and Ce_0.87_Ln_0.13_O_1.935_ phases, in order to estimate the mean (volume average) crystallite size (*D*) by applying the Scherrer equation, with a correction for instrument line broadening using quartz as pattern.

#### 2.3.3. Textural Characterization

The textural characterization of both the fresh and thermally aged samples was accomplished by physical adsorption of N_2_ at −196 °C using an automatic Autosorb iQ_3_ equipment (Quantachrome, Boynton Beach, FL, USA) and working at relative pressures (*p*/*p*^0^), ranging from 0.1 to 1.0. Prior to starting the adsorption-desorption measurements under equilibrium conditions, about 100 mg of powder sample was out-gassed under vacuum at 200 °C for 12 h, in order to remove moisture and any possible gases and vapours from the laboratory atmosphere adsorbed on the material surface. The measured N_2_ adsorption-desorption isotherms provided valuable information concerning the pore size distribution in the micro- and mesoporosity regions, surface area, and pore volume. The pore size distribution curves in the mesopore range were estimated by applying the method developed by Barrett, Joyner and Halenda (BJH) [50] to the desorption branch of the experimental isotherms. The apparent surface areas (*S*_BET_) were assessed by the Brunauer, Emmett and Teller (BET) equation [51], which was as a rule applied in the *p*/*p*^0^ range from 0.05 to 0.20. Moreover, the total pore volumes (*V*_p_) were calculated from the volumes of N_2_ adsorbed at a *p*/*p*^0^ value of 0.99, expressed as the corresponding liquid volumes.

#### 2.3.4. Surface Chemical Characterization

X-ray photoelectron spectroscopy (XPS) analyses were carried out on a Kratos Axis Ultra^DLD^ spectrometer (Kratos Analytical Ltd., Manchester, UK), using monochromatized Al K*α* radiation (*hν* = 1486.6 eV), with a selected X-ray power of 150 W. High resolution spectra were collected under the CAE (constant analyser energy) mode, with a pass energy of 20 eV. Fresh and thermally aged samples, without any further treatment, were compressed into self-supported pellets and then mounted on the sample holder, by means of a double-sided adhesive conducting carbon tape. The coaxial charge neutralization system developed by Kratos was employed to compensate surface charging effects. The binding energy (*BE*) scale was calibrated with respect to the C 1*s* signal, coming from adventitious carbon contamination, and set at 284.8 eV, according to the literature [52]. CasaXPS software (version 2.3.19rev1.1m, Casa Software Ltd., Devon, UK) was used for spectra processing.

#### 2.3.5. Electron Microscopy Characterization

Both the freshly prepared and thermally aged samples were also characterized at the micro- and nanoscale by a set of electron microscopy techniques, including high resolution electron microscopy (HREM), high angle annular dark field-scanning transmission electron microscopy imaging (HAADF-STEM), and energy-dispersive X-ray spectroscopy (X-EDS). These analyses were carried out in a Talos F200X scanning transmission electron microscope (FEI, Thermo Scientific, Waltham, MA, USA), equipped with a high efficiency X-EDS ChemiSTEM system. Previously, small amounts of the powdered materials, as prepared without any additional treatment, were deposited onto holey carbon-coated TEM grids.

#### 2.3.6. Reducibility Measurements

The redox behaviour of both the fresh and thermally aged materials was studied by the temperature-programmed reduction (TPR) technique followed by mass spectrometry (MS). TPR-MS experiments were conducted in a conventional device coupled to a quadrupole mass spectrometer (Thermostar GSD301T1, Pfeiffer Vacuum, Wetzlar, Germany), in order to analyse the composition of the outlet gas stream. The amount of sample typically employed in each set of essays was around 150 mg. All three fresh nanostructured materials were subjected to the same above-described sequence of thermochemical ageing treatments, with the recording of the MS signal during the consecutive reduction steps carried out at increasing temperatures from 500 to 900 °C. The main mass/charge (m/z) ratios registered during these experiments were 2 (H_2_^+^) to monitor hydrogen consumption and 18 (H_2_O^+^) for the concomitant evolution of water. At this point, it should be highlighted that TPR results are herein depicted as water evolution curves, a complete consistency with hydrogen consumption profiles being observed for all tested materials. Additionally, the m/z ratios 28 (CO^+^), 30 (NO^+^), 40 (Ar^+^), and 44 (CO_2_^+^ and N_2_O^+^) were also followed.

## 3. Results and Discussion

### 3.1. Characterization of the Fresh Undoped and Ln-Doped Nanostructured ZrO_2_-CeO_2_ Samples

The freshly prepared undoped and Ln-doped nanostructured ZrO_2_-CeO_2_ samples were analysed by the XRF technique, in order to estimate their actual Ce and Ln contents. Obtained quantification data are listed in Table 1. From these results, it becomes apparent that both the CeO_2_ and Ln-doped CeO_2_ molar loadings were very close to the nominal value of 15 mol.%, being slightly higher for the undoped sample (i.e., 16.6 mol.%) and somewhat lower for their Ln-doped counterparts (14.2 and 13.5 mol.% for the Y- and La-containing materials, respectively). Furthermore, the dopant to cerium atomic ratio was 14.2% for yttrium and 14.6% for lanthanum, well in agreement with the expected value of 15%. Therefore, it may be concluded that, at least as far as the overall macroscopic chemical composition of the resulting nanomaterials is concerned, the employed surfactant-free wet chemistry route was successful and achieved the starting synthetic goal.

The analysis of surface chemical features is a key issue regarding the characterization of heterogeneous catalysts, and particularly of those comprising supported oxide phases, like the nanostructured ZrO_2_-CeO_2_ materials investigated in the present work. Table 2 gathers the quantitative data derived from the X-ray photoelectron spectra for all three fresh samples. In this regard, it should be noted that, for such a quantification purpose, only those XPS signals originating from similar depths (i.e., Zr 3*d*, Ce 4*d*, Y 3*d*, and La 4*d* photoelectrons, whose inelastic mean free path (IMFP) values are 2.10, 2.18, 2.12, and 2.18 nm, respectively, when travelling through a CeO_2_ layer [53]) have been considered. Moreover, the cerium and rare earth dopant to zirconium atomic ratios (i.e., (Ce + Ln)/Zr) assessed from XRF measurements, which stand for the average bulk composition of the samples, have also been included in the above table for the sake of comparison. These latter values have been taken herein as references with a view to estimate the integration degree of CeO_2_-based nanoclusters into the ZrO_2_ support, after applying the different thermochemical ageing treatments, as will be further explained below.

From the above results, it is clearly seen that the (Ce 4*d* + Ln X*d*)/Zr 3*d* ratios for the fresh nanocatalyst samples are markedly higher than the corresponding (Ce + Ln)/Zr nominal values. This observation unambiguously corroborates the essentially surface character of CeO_2_-based nanoclusters, as well as the efficiency of the proposed synthetic approach.

**Figure 2:**
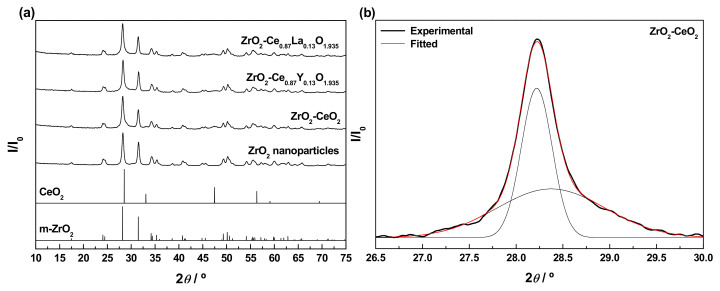
(**a**) XRD patterns recorded for the commercial ZrO_2_ nanoparticles and the fresh undoped and Ln-doped ZrO_2_-CeO_2_ samples, and (**b**) fitted and experimental diagrams of the diffraction peak, centred at around 28.2° for the fresh ZrO_2_-CeO_2_ nanocomposite.

The XRD patterns collected for the bare nanoparticulated ZrO_2_ support and the prepared undoped and Ln-doped ZrO_2_-CeO_2_ nanomaterials are depicted in Figure 2a. For comparison purposes, the diffractograms corresponding to pure monoclinic ZrO_2_ and cubic fluorite CeO_2_ (JCPDS card numbers 00-037-1484 and 34-0394, respectively) have also been included in the above figure. The pattern for the ZrO_2_ support only shows the characteristic reflections associated with the monoclinic phase (space group *P*2_1_/*c*), thus allowing one to exclude the coexistence of tetragonal and monoclinic phases, typically found for ZrO_2_ samples synthesized by a variety of methods and procedures [54,55,56]. The average crystallite size, as estimated by applying the Scherrer equation, was around 23.4 nm, well in agreement with the mean diameter within the range from 20 to 30 nm provided by the supplier for these commercial ZrO_2_ nanoparticles. After the deposition of the CeO_2_-based surface phases, no diffraction peaks ascribable to crystalline CeO_2_ or Ln-doped CeO_2_ with cubic fluorite-type structure (space group *Fm*-3*m*) are clearly distinguishable in the diagrams of the resulting nanocomposite samples. This fact may be accounted for, on the one hand, by the relatively low content of these CeO_2_-containing phases in the prepared nanocatalysts and, on the other, by their highly dispersed state on the surface of the ZrO_2_ support and very small crystallite sizes, likely below the detection limit of the XRD technique (i.e., 4–5 nm) [57]. In this regard, it should be highlighted that the amount of Ln^3+^ dopants incorporated into the lattice of the CeO_2_ surface phase (i.e., around 15 at.%) to yield the corresponding solid solutions is far below their solubility limits, which have been estimated to be ca. 20 and 52 at.% for Y^3+^ and La^3+^, respectively [58,59,60]. Therefore, it was expected that both dopants were effectively integrated into the CeO_2_ lattice and not as segregated crystalline phases of pure Ln_2_O_3_ or Ln^3+^-rich mixed oxide. By contrast, in these patterns, the main peaks of the monoclinic ZrO_2_ remain still well-defined and visible, but obviously somewhat weakened as compared to their counterparts in the diffractogram for the raw ZrO_2_ nanoparticles. As the only distinctive feature, the (111) diffraction peak of monoclinic ZrO_2_ centred at around 28.2° exhibits a slight asymmetry in the form of a tail to the right, being more evident for the undoped ZrO_2_-CeO_2_ sample. Such a characteristic is attributable to its overlap, with the most intense reflection of fluorite-type CeO_2_ appearing at approximately 28.5° and corresponding to the (111) crystallographic planes. In fact, the peak at 28.2° in the experimental XRD diagram for the fresh ZrO_2_-CeO_2_ nanocatalyst can be deconvoluted into two Gaussian peaks located at around 28.2° and 28.4° (see Figure 2b for more details); this latter being much less intense and broader than the former due to the smaller crystallite size of the CeO_2_ phase in comparison to the ZrO_2_ nanoparticles. At this point, it is worth mentioning that the effective incorporation of Ln^3+^ dopants into the CeO_2_ cubic lattice to form a solid solution entails a modification of the cell parameter (*a*_0_), and thereby a certain displacement of its characteristic reflections, whose magnitude strongly depends on the relative size of the foreign cation with respect to the host Ce^4+^, as well as on the dopant concentration. Thus, for smaller cations such as Y^3+^, the cell parameter is reduced and the fluorite diffraction peaks are slightly shifted to higher 2*θ* angles, while the opposite applies to larger dopants like La^3+^ [58,59]. Accordingly, such an effect should be kept in mind, with a view to analysing the position of the (111) main reflection of the cubic fluorite-type CeO_2_ phase in the XRD diagrams for the Ln-doped materials, as compared to that observed for the ZrO_2_-CeO_2_ reference sample. However, a clear identification of this diffraction peak is seriously hindered by its width, consequence of the small crystallite size, and overlap with the very intense (111) reflection of monoclinic ZrO_2_ at ca. 28.2°. Furthermore, from the registered patterns, the formation of any ZrO_2_-CeO_2_ mixed oxide crystalline phase as a result of the diffusion of the deposited CeO_2_-containing nanocrystallites inside the lattice of the ZrO_2_ support during the calcination treatment at 350 °C in air can be completely discarded. In brief, all these observations seem to be compatible with the presence in the prepared nanocomposite samples of CeO_2_-based surface phases, consisting of crystallites or clusters with nanometric dimensions in a high dispersion degree and covering the ZrO_2_ nanoparticles. As previously pointed out, this surface nature of the undoped and Ln-doped CeO_2_ phases is also corroborated by the elemental chemical compositions for the outermost atomic layers derived from XPS analyses (see Table 2 again).

**Figure 3:**
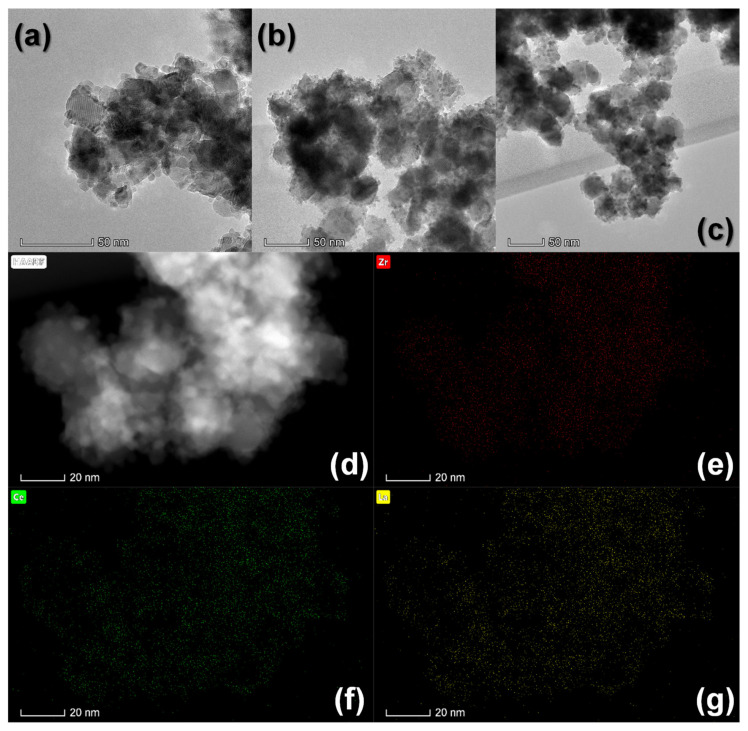
Representative TEM images for the fresh samples: (**a**) ZrO_2_-CeO_2_, (**b**) ZrO_2_-Ce_0.87_Y_0.13_O_1.935_, and (**c**) ZrO_2_-Ce_0.87_La_0.13_O_1.935_. (**d**) High angle annular dark field-scanning transmission electron microscopy imaging (HAADF-STEM) image of a selected area of the fresh ZrO_2_-Ce_0.87_La_0.13_O_1.935_ sample and the corresponding X-EDS maps for (**e**) Zr (red), (**f**) Ce (green), and (**g**) La (yellow).

The aforesaid morphological features are corroborated from the TEM images registered for the fresh undoped and Ln-doped ZrO_2_-CeO_2_ samples and gathered in Figure 3a–c. Additionally, a representative medium-magnification HAADF-STEM image of a selected area of the fresh ZrO_2_-Ce_0.87_La_0.13_O_1.935_ nanocatalyst is also shown in Figure 3d. Because, in this imaging mode, the intensity increases with both the thickness and atomic number of the elements in the imaged area (with a dependence of the type *Z*^1.8^), the above figure clearly suggests that the nanosized particles supported on the surface of the larger and round-shaped ZrO_2_ crystals are essentially composed of the heavier elements Ce and La. In any case, the presence of these Ce and La-containing nanocrystals is further evidenced by the corresponding X-EDS maps, which are depicted in Figure 3e–g. From these elemental distribution maps, it becomes evident that the supported nanosized particles exhibit a much higher concentration of both Ce and La as compared to Zr, thus consisting of La-doped CeO_2_. Very similar results are obtained for fresh ZrO_2_-CeO_2_ and ZrO_2_-Ce_0.87_Y_0.13_O_1.935_ nanocatalyst samples, so their HAADF-STEM images and X-EDS maps have been omitted for the sake of brevity.

**Figure 4:**
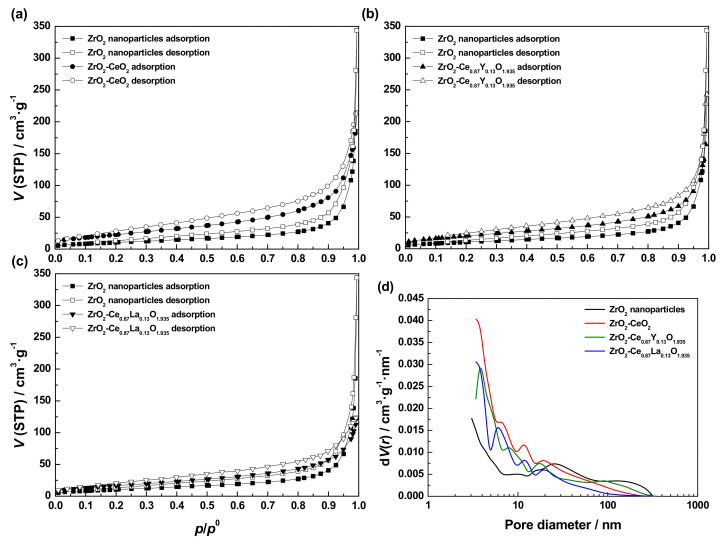
(**a**–**c**) Nitrogen adsorption-desorption isotherms at −196 °C and (**d**) BJH pore size distributions for the commercial ZrO_2_ nanoparticles and the fresh undoped and Ln-doped ZrO_2_-CeO_2_ samples.

The N_2_ adsorption-desorption isotherms collected for the pristine ZrO_2_ nanoparticles and the freshly prepared undoped and Ln-doped ZrO_2_-CeO_2_ nanocomposite samples are plotted together in Figure 4, for the sake of comparison. Such isotherms by their shape closely resemble a combination of types II and IV of the recently updated IUPAC classification system [61], so that these nanostructured oxides can be considered as essentially mesoporous materials with negligible microporosity. In this regard, the absence of micropores in the nanocomposite samples is clearly evidenced by the very low adsorption of N_2_ at *p*/*p*^0^ values below 0.1.

In general terms, the N_2_ adsorption-desorption isotherms measured for the undoped and Ln-doped ZrO_2_-CeO_2_ systems are rather similarly shaped to that registered for the bare ZrO_2_ nanoparticles support. As the only prominent difference, the amount of adsorbed N_2_ over the entire *p*/*p*^0^ range is notably greater for the fresh nanocomposite samples. Therefore, it becomes apparent that the deposition of the CeO_2_-based nanoclusters brings about a significant porosity development and thereby an increase in specific surface area. This conclusion is confirmed from the textural parameters estimated for the ZrO_2_ support and the synthesized nanostructured oxide samples and gathered in Table 3. Such an increase in porosity and surface area may be essentially attributed to nanometer-sized CeO_2_-containing crystallites or clusters (i.e., below 4–5 nm in size, as inferred from XRD measurements) highly dispersed on the surface of larger ZrO_2_ nanoparticles, thus leading to surface roughness and porosity in the resulting composite materials.

As far as the influence of the Ln^3+^ dopants on the textural features is concerned, from the data listed in Table 3, it is clearly observed that their incorporation into the supported CeO_2_-based phase has a detrimental effect on the specific surface area (*S*_BET_) values for the resulting nanocomposite materials in comparison to the ZrO_2_-CeO_2_ sample, taken as reference. Such a decrease is by far much more pronounced for the La^3+^-doped ZrO_2_-CeO_2_ nanomaterial, i.e., a 25% of loss in surface area versus a 10% for its Y^3+^-containing counterpart. Moreover, from Figure 4d, it follows that the pore size distribution of the ZrO_2_-CeO_2_ sample is also slightly affected by the doping with both Ln^3+^ cations, especially in the region corresponding to the smallest size pores and for the Y^3+^-doped nanocatalyst. By contrast, the mean pore diameter (*D*_p_) appears to remain nearly unaltered and within the mesoporosity range (i.e., from 3 to 4 nm) for all the three fresh samples. However, at this point, it is worth mentioning that most of the meso- and macroporosity observed in the above curves is very likely to belong to gaps or voids between closely spaced grains and particles, forming aggregates of the prepared nanocomposites.

**Figure 5:**
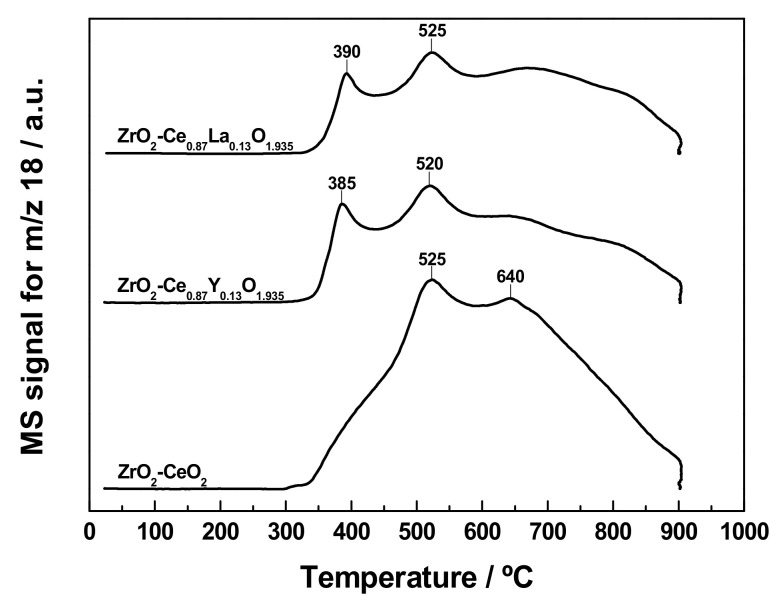
TPR-MS profiles registered for the fresh undoped and Ln-doped ZrO_2_-CeO_2_ samples.

The reduction behaviour of the freshly prepared undoped and Ln-doped ZrO_2_-CeO_2_ nanocomposite samples was investigated by means of the TPR-MS technique under a H_2_ (5%)/Ar atmosphere after a standard cleaning pre-treatment in O_2_ (5%)/He at 350 °C for 1 h. For the sake of comparison, the recorded curves have been plotted together in Figure 5. First, it should be borne in mind that ZrO_2_ is a very stable oxide, without any reducibility under the conditions employed in these experiments, so it is expected that the different features displayed by the above traces are entirely due to the supported CeO_2_-based surface phases. In order to confirm this, a TPR-MS experiment was also carried out on the bare ZrO_2_ nanoparticles used as support, the obtained profile being depicted in Figure S1. As the only prominent features, it exhibits a broad and very low intensity H_2_O evolution signal (i.e., between one and two orders of magnitude lower than those recorded for the ZrO_2_-CeO_2_ nanocatalysts), covering the range from 400 to 900 °C, with a couple of peaks at roughly 530 and 670 °C, the latter being sharper and much more intense than the former. Since the first H_2_O evolution peak is also accompanied by CO release, it is compatible with the reduction of a variety of carbonate species chemisorbed on the surface of the ZrO_2_ nanoparticles and not decomposed in the oxidizing treatment previous to the TPR run. Concerning the sharper peak at higher temperature, it may be tentatively ascribed to evolved H_2_O coming from the dehydroxylation of the ZrO_2_ surface.

As far as the profile for the fresh ZrO_2_-CeO_2_ sample is concerned, only two major reduction events are clearly observed. These are a relatively sharp and intense peak centred at around 525 °C with a greatly sloped shoulder at ca. 465 °C, followed by a very broad band extending up to 900 °C with a maximum located at roughly 640 °C. The former event entails both H_2_ consumption and H_2_O evolution (readers are referred to Figure S2a in the Supplementary Material), so it can be unambiguously ascribed to oxygen abstraction from the nanocomposite sample. Particularly, this feature is associated with the surface reduction of CeO_2_ nanoclusters supported on ZrO_2_ nanoparticles. Under a H_2_ (5%)/Ar reducing atmosphere, and for a number of pure CeO_2_ powdered and nanostructured materials, such process has been widely reported to start at temperatures slightly below 400 °C, reaching its maximum in the range from 500 to 600 °C [62,63,64,65,66,67]. By contrast, the very broad band peaked at ca. 640 °C involves H_2_ consumption, as well as evolution, not only of H_2_O, but also of CO (see Figure S2a in the Supplementary Material). Accordingly, this second event in the TPR profile cannot be exclusively attributed to the bulk reduction of CeO_2_ nanoclusters, which is well-known to occur at around 830 °C [62,63]. In addition, it also contains a notable contribution coming from the reduction of those carbonate species strongly bonded to both the CeO_2_ crystallites and the ZrO_2_ support, and not removed during the pre-treatment at 350 °C in oxidizing conditions. Therefore, it may be concluded that the aforesaid broad band in the water trace results from the overlap of several reduction processes, involving the bulk of supported CeO_2_ nanoclusters and residual carbonates. At this point, it is worth noting that, for pure CeO_2_ samples, the intensity of the low-temperature reduction peak relative to that of its high-temperature counterpart strongly depends on the surface of the materials, being obviously greater for those with larger surface area values. The very low mean crystallite size (i.e., below 4–5 nm) estimated for the supported CeO_2_ phase brings about a large surface area to volume ratio and thereby an enhanced fraction of cerium and oxygen atoms at the surface, with respect to the total amount of atoms in the crystallite, which would account for the more intense surface reduction peak dominating the water evolution profile for the ZrO_2_-CeO_2_ sample. In fact, a dramatic increase in the surface reduction peak at the expense of the bulk reduction one with decreasing particle size has been previously reported and quantified for a series of CeO_2_ samples, with mean particle sizes ranging from 4 to 13 nm [64], as well as for CeO_2_ highly dispersed on a commercial SiO_2_ support [68]. In the latter case, the authors noticed that an inversion of the intensity of the two reduction peaks took place for CeO_2_ crystallites, having sizes of around 5 nm and below. Finally, it should be also highlighted that this TPR diagram obtained on the fresh ZrO_2_-CeO_2_ sample closely resembles those earlier reported by our research group for ZrO_2_- and YSZ-supported CeO_2_ catalyst formulations, containing similar lanthanoid oxide nominal loadings (i.e., 15 and 13 mol.%, respectively), and prepared by the incipient wetness impregnation method [38,39]. In any case, all these three TPR profiles clearly evidence the effectiveness of the proposed synthetic approach, consisting of depositing CeO_2_ in the form of a surface phase, either by incipient wetness impregnation or by the controlled chemical precipitation method herein described, to improve the redox response of the resulting ZrO_2_-CeO_2_ systems when compared with a commercial fresh bulk mixed oxide sample with similar Ce to Zr molar ratio (i.e., Ce_0.15_Zr_0.85_O_2_) and reported elsewhere [38,39].

Regarding the fresh Ln-doped samples, their TPR profiles are fairly well similarly shaped irrespective of the Ln^3+^ dopant, and bear great resemblance to the curve registered for the undoped nanocomposite material. Thus, the former profiles display the rather intense peak accompanied by the very broad band located at nearly the same temperatures (i.e., 520 and 640 °C for the Y^3+^-doped nanocatalyst, and 525 and 670 °C for the La^3+^-containing one), that are observed as well in the water trace of the ZrO_2_-CeO_2_ reference sample. In principle, the tentative assignment of these features is essentially the same than that discussed above for the fresh undoped material: the peak is related with the surface reduction of the supported CeO_2_-based nanoclusters, whereas the band at higher temperature encompasses the reduction both of the bulk of these CeO_2_ nanoclusters and the residual carbonates. Concerning these latter species, both their amount and stability are expected to be slightly higher for the La^3+^-modified nanocatalyst than for the undoped material, chiefly due to the more basic character of La^3+^ in comparison with Ce^3+^ and Ce^4+^, while the opposite should apply to the sample containing the less basic Y^3+^ dopant [69,70]. On the other hand, the only remarkable difference between the TPR curves for the reference and Ln-doped ZrO_2_-CeO_2_ samples arises from the quite intense and sharp peak appearing at around 385–390 °C for the latter materials. As inferred from Figures S2 and S3 in the Supplementary Material, such a feature corresponds to a reduction event involving H_2_ consumption and evolution of H_2_O, together with CO and NO. Accordingly, it may be at least partly assigned to the reduction of residual nitrate species remaining in the freshly prepared Ln-modified samples, after the calcination step in air at 350 °C for 2 h. Notwithstanding, the larger contribution to this low temperature peak is likely to come from the improvement in the reducibility of the supported CeO_2_ nanoclusters, as a consequence of the vacancies created in the oxygen sub-lattice, by the incorporation of both Ln^3+^ dopants [44,71]. In this connection, similar peaks have been earlier reported in the water evolution traces collected during TPR experiments for bulk CeO_2_-Y_2_O_3_ and CeO_2_-La_2_O_3_ mixed oxides, with varying compositions [60,71].

**Figure 6:**
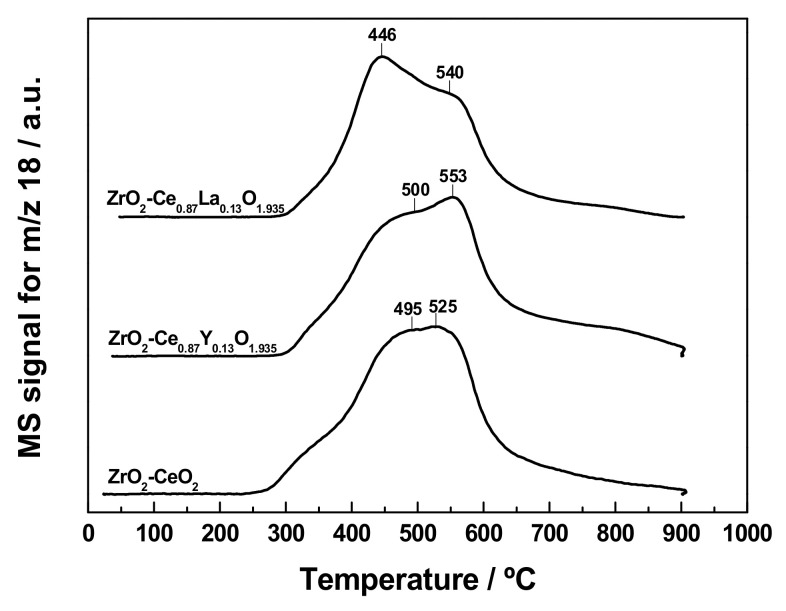
Temperature-programmed reduction mass spectrometry (TPR-MS) profiles registered for the undoped and Ln-doped ZrO_2_-CeO_2_ samples previously subjected to a reduction treatment in H_2_ (5%)/Ar, at 900 °C for 1 h.

**Figure 7:**
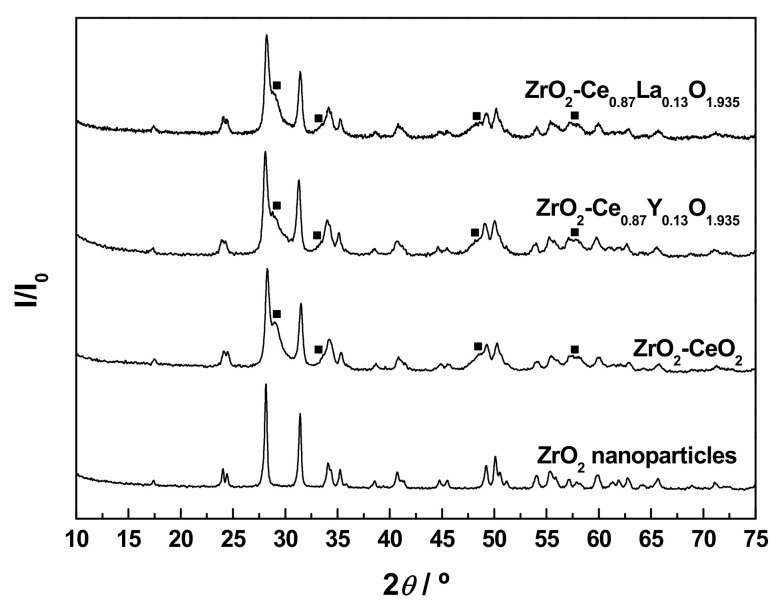
XRD patterns recorded for the undoped and Ln-doped ZrO_2_-CeO_2_ samples subjected to a reduction treatment in H_2_ (5%)/Ar at 900 °C for 1 h. Caption: ■ Ce_x_Zr_1−x_O_2−δ_ phases.

In order to shed light on the chemical modifications induced by the reduction treatment at 900 °C for 1 h, as well as on their effects on the redox properties, the thermally aged samples resulting from the first TPR run were further subjected to a second successive one, under identical experimental conditions and following the same protocol. The registered H_2_O evolution profiles are gathered in Figure 6. From this figure, it becomes evident that the curves for the thermally aged nanocatalysts are markedly different in comparison with those obtained for their fresh counterparts and shown in Figure 5, regardless of whether they are doped or not, and of the nature of the Ln^3+^ dopant. Indeed, all the three profiles are dominated by a very broad band, encompassing the 300 to 650 °C range, which apparently seems to be the result of the overlap of at least three major reduction events, and probably more, occurring over different temperature intervals (notice that the H_2_O signals are exclusively accompanied by H_2_ uptake, so they can be unambiguously ascribed to reduction processes). These are two main peaks centred at ca. 445 to 500 °C and 525 to 555 °C, and a greatly sloped shoulder at around 360 to 375 °C. The latter feature is much more evident for the undoped reference sample and poorly visible for the doped nanocatalysts, particularly for the La-modified one. Furthermore, the incorporation of the Ln^3+^ dopant also appears to strongly alter the relative intensity of the aforementioned peaks. Thus, while they display almost the same intensity in the case of the ZrO_2_-CeO_2_ material, the first peak is more intense than the second one for the La-doped sample, and the opposite applies to that containing Y. In any case, these TPR profiles in Figure 6 bear a striking resemblance to those previously reported in the literature for a number of bulk cerium-zirconium mixed oxides (Ce_x_Zr_1−x_O_2_), with varying compositions [72,73], thereby suggesting that the formation of a ZrO_2_-CeO_2_ solid solution in the three prepared samples could occur, as expected, during the first reduction treatment in H_2_ (5%)/Ar, at 900 °C for 1 h. As evidenced from Figure 7, such an assumption is corroborated by the appearance of some additional reflections in the XRD patterns collected for the reduced samples, resulting from the first TPR run with respect to those registered for the fresh nanocatalysts (cf. Figure 2). These additional reflections include a few very broad peaks centered at around 29.0°, 33.7°, 48.5°, and 57.4°, thus suggesting a very small average crystallite size for the corresponding phases. Moreover, as a rule, they are rather weak and badly visible, due to their overlap with the dominating and well-defined diffraction peaks of the monoclinic ZrO_2_ support, except for the reflection located at ca. 29.0°, which is the most intense, and thereby the only readily visible. This set of diffraction peaks advocates for the formation of at least a Ce_x_Zr_1−x_O_2−δ_ phase, and probably more or even a gradient of them, after the heavy reduction treatment at 900 °C. In this regard, it is well-known that the insertion of Zr^4+^ cations (*r* = 92 pm [43]), smaller than both Ce^4+^ and Ce^3+^ (*r* = 111 pm and 128 pm, respectively [43]), into the fluorite-type structure of CeO_2_ or partially reduced CeO_2_ (i.e., CeO_2−δ_) to form solid solutions, induces a shrinkage of the cubic cell [3], and hence, a shift of its characteristic reflections to higher 2*θ* angles, whose extent increases with the position of the peak. Accordingly, the displacements observed for the main diffraction peaks of fluorite CeO_2_ from 28.5°, 33.1°, 47.5°, and 56.3° to ca. 29.0°, 33.7°, 48.5°, and 57.4°, respectively, in the XRD patterns for the nanocatalyst samples reduced at 900 °C (see Figure 7), appear to be well in agreement with the formation of several Ce_x_Zr_1−x_O_2−δ_ phases during the reduction treatment. Unfortunately, the precise compositions, crystalline structures, and lattice parameters of such mixed oxide phases are very difficult to accurately identify exclusively from XRD measurements, even by applying the Rietveld analysis to the experimental diffractograms. This identification problem is exacerbated by the fact that the XRD diagrams were recorded for the reduced samples previously subjected to passivation, thus ensuring the presence of both Ce^3+^ and Ce^4+^ cations in an undefined proportion and leading to more complex Ce_x_Zr_1−x_O_2−δ_ phases. Nonetheless, it is worth highlighting that the abovementioned family of new reflections is attributable neither to the sintering of the supported CeO_2_-based nanoclusters during the thermochemical treatment at 900 °C under reducing atmosphere, nor to a CeO_2−δ_ phase retaining a cubic fluorite structure. In the former case, the size enlargement of the CeO_2_ crystallites would have also led to the appearance in the TPR profiles of the very broad band at high temperatures associated with the bulk reduction of this oxide, while in the latter case, the typical fluorite diffraction peaks would have shifted to lower 2*θ* values, due to the presence of some larger Ce^3+^ cations in the CeO_2_ lattice.

On the other hand, the formation of mixed oxide surface phases is also supported by the quantitative results derived from the XPS analyses performed on the undoped and Ln-doped ZrO_2_-CeO_2_ nanocatalysts after applying the reduction treatment at 900 °C, which are set out in Table 4. From these data, it is evident that the (Ce 4*d* + Ln X*d*)/Zr 3*d* ratio markedly drops for the heavily reduced samples in comparison with those estimated for the fresh materials (see Table 2), irrespective of the Ln^3+^ dopant. This behaviour may be accounted for either the aforesaid growth of the surface CeO_2_-containing nanoclusters or the formation of Ce_x_Zr_1−x_O_2−δ_ surface phases. Again, the TPR profiles shown in Figure 6 allow one to consider this second explanation as the most feasible one. Notwithstanding, it should be noted that the ratios estimated by XPS (surface composition) are still considerably higher than the corresponding atomic ratios obtained by the XRF technique (bulk composition), thus confirming that the formation of the mixed oxide phases took place only down to a certain depth in the ZrO_2_ nanoparticle support.

Once the presence of cerium-zirconium mixed oxide surface phases in the nanocatalyst samples reduced at a high temperature has been postulated, an essential question arising is whether their formation occurs in a progressive way during the application of the reduction treatment, or if, on the contrary, a minimum temperature is required for the integration between supported CeO_2_ nanoclusters and ZrO_2_ nanoparticles to reach a significant degree. The following section will be devoted to studying this question in detail, as well as to clarifying the influence of the incorporation of small amounts of non-reducible Ln^3+^ cations with different sizes.

### 3.2. Characterization of the Thermally Aged Undoped and Ln-Doped ZrO_2_-CeO_2_ Samples

In order to address the study of the above question, the freshly prepared undoped and Ln-doped ZrO_2_-CeO_2_ materials were subjected to the series of successive thermochemical ageing treatments previously described in Section 2.2, and schematically illustrated in Figure 1. Then, the thermally aged samples resulting from each reduction treatment, after applying the appropriate passivation protocol, were carefully characterized, in terms of their texture, structure, surface chemical composition, and redox properties.

#### 3.2.1. Textural Characterization

**Figure 8:**
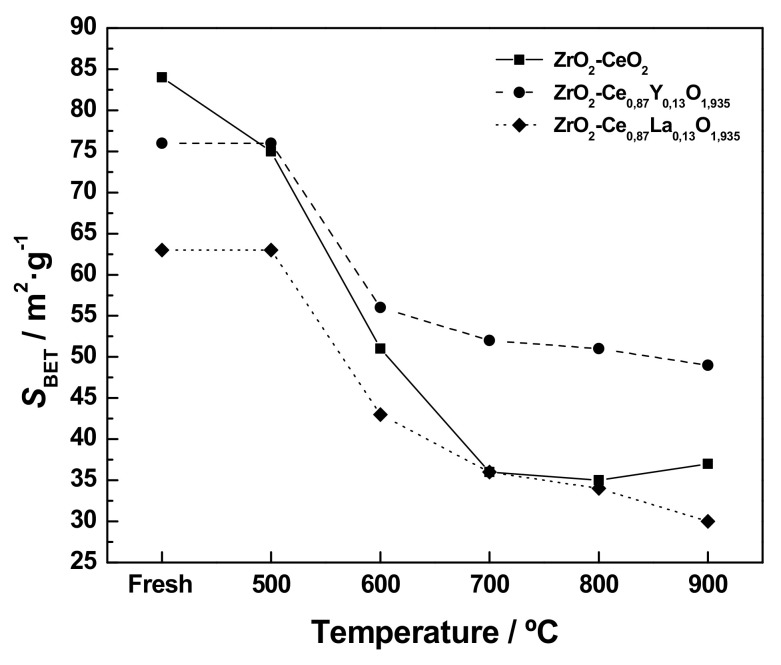
Evolution of *S*_BET_ with the maximum temperature of the last reduction treatment for the undoped and Ln-doped ZrO_2_-CeO_2_ samples during the application of the successive thermochemical ageing treatments.

For the sake of brevity, the N_2_ adsorption-desorption isotherms registered for the thermally aged nanocatalyst samples have been omitted, since they are very similarly shaped to those measured for the fresh nanomaterials and gathered in Figure 4a–c. Specifically, the only relevant modification concerns the amount of adsorbed N_2_, due to the different surface area of the samples submitted to the thermochemical ageing treatments. Figure 8 depicts the evolution of the specific surface area (*S*_BET_) values for the undoped and Ln-doped ZrO_2_-CeO_2_ nanocatalysts, with the maximum temperature of the last applied reduction treatment. As expected, such treatments are accompanied by a strong loss of surface area for all the three prepared samples, regardless of the Ln^3+^ dopant, the decrease being much more pronounced up to a reduction temperature of 700 °C. In stark contrast, the subsequent reduction treatments at higher temperatures hardly modify the surface area of the resulting thermally aged nanocatalysts. This variation trend may be attributed to a combined effect of two major processes: the sintering of the CeO_2_-based nanoclusters supported on the ZrO_2_ nanoparticles as the temperature rises during the successive reduction treatments, on the one hand, and the formation of a cerium-zirconium mixed oxide surface phase, on the other. The former process is likely predominant at mild reduction temperatures, whereas the latter one could dominate under severe reducing conditions. Furthermore, it should be pointed out that the aforesaid surface loss effect is notably lower for the Y-containing nanocatalyst, with respect to its undoped and La-doped counterparts. In fact, while the decrease in surface area for these two samples after applying the series of consecutive thermochemical treatments is estimated to be around 56.0 and 52.4%, respectively, this value markedly drops up to 35.5% for the Y-modified material. Such an apparent inhibition of sintering as a consequence of the incorporation of yttrium into the lattice of the supported CeO_2_ phase has been previously reported in the literature [19] and tentatively associated with the segregation of this rare earth element at crystallite boundaries [74,75,76], thus enhancing the thermal stability. Finally, it is also worth noting that the *S*_BET_ values obtained for the heavily reduced samples (i.e., those heat-treated at 900 °C, the highest reduction temperature applied in the present work) are very close to that for the ZrO_2_ nanoparticles support (i.e., ca. 35 m^2^·g^−1^).

#### 3.2.2. Structural Characterization

**Figure 9:**
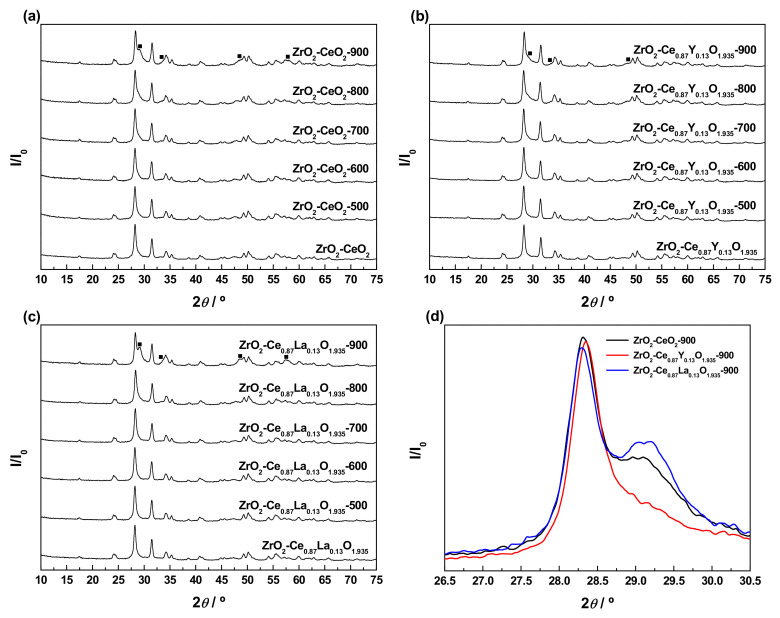
(**a**–**c**) XRD patterns recorded for the undoped and Ln-doped ZrO_2_-CeO_2_ samples, subjected to successive thermochemical ageing treatments at increasing reduction temperatures from 500 to 900 °C. (**d**) Enlargement of the (111) diffraction peak of monoclinic ZrO_2_ for the undoped and Ln-doped ZrO_2_-CeO_2_ samples subjected to the whole series of reduction treatments up to 900 °C. Caption: ■ Ce_x_Zr_1__−__x_O_2__−δ_ phases.

An in-depth characterization study was conducted, in order to follow and understand the evolution of the structural features of both the undoped and Ln-doped nanocatalysts with the successive thermochemical ageing treatments, at increasing reduction temperatures from 500 to 900 °C. The diffractograms were recorded for the thermally aged samples resulting from each reduction treatment after the appropriate passivation, all of them being gathered in Figure 9a–c. For comparison purposes, the XRD patterns registered for the freshly prepared materials have also been included in the above plots. As clearly seen, after applying the series of thermochemical ageing treatments, the whole set of diagrams is still dominated by the very intense and sharp reflections typical of the monoclinic ZrO_2_ employed as support, irrespective of whether the samples are doped or not and of the Ln^3+^ dopant. Apart from subtle modifications affecting their position and narrowing, such diffraction peaks are readily visible, thus revealing that both the structure and average size of the ZrO_2_ crystallites remain essentially unaltered. As the only remarkable changes, the consecutive thermochemical treatments lead to the appearance of a few weak and very broad reflections located at around 29.0°, 33.7°, 48.5°, and 57.4°, which are by far much more evident for the samples aged at the highest reduction temperature (i.e., ZrO_2_-CeO_2_-900, ZrO_2_-Ce_0.87_Y_0.13_O_1.935_-900, and ZrO_2_-Ce_0.87_La_0.13_O_1.935_-900). The positions and relative intensities of these diffraction peaks are in very good agreement with those previously detected in the XRD diagrams for the undoped and Ln-doped nanocatalysts submitted to a reduction treatment at 900 °C for 1 h under H_2_ (5%)/Ar atmosphere (cf. Figure 7), which were ascribed to the presence of several cerium-zirconium mixed oxide surface phases. According to the patterns shown in Figure 9a–c, the formation of these phases apparently starts to be significant (i.e., detectable by means of the XRD technique) after the application of the reduction treatment at 700 °C and, especially, at 800 °C.

As far as the effect of the incorporation of the Ln^3+^ dopants into the supported CeO_2_ phase is concerned, the enlargement of the 2*θ* range corresponding to the (111) planes of the monoclinic ZrO_2_ support, between 26.5° and 30.5°, has been depicted in Figure 9d, for the three materials subjected to the whole series of reduction treatments up to 900 °C. From these plots, it becomes evident that the relative intensity of the shoulder appearing at ca. 29.0°, which in a first approach may be regarded as indicative of the amount of mixed oxide phases formed in the thermally aged samples, is noticeably higher for the La-doped nanocatalyst in comparison with the reference ZrO_2_-CeO_2_ material, while the opposite applies to its Y-modified counterpart. Therefore, exclusively from XRD measurements, it may be postulated that the introduction of La^3+^ promotes the effective integration between the supported CeO_2_-based nanoclusters and the ZrO_2_ nanoparticles support to yield several mixed oxide surface phases, whereas the presence of Y^3+^ seems to have a detrimental effect and inhibits, to some extent, such an integration process. A later subsection will be focused on corroborating this conclusion, by accomplishing a detailed analysis of the compositional changes at the surface for the thermally aged samples by means of the XPS technique.

#### 3.2.3. Redox Behaviour

**Figure 10:**
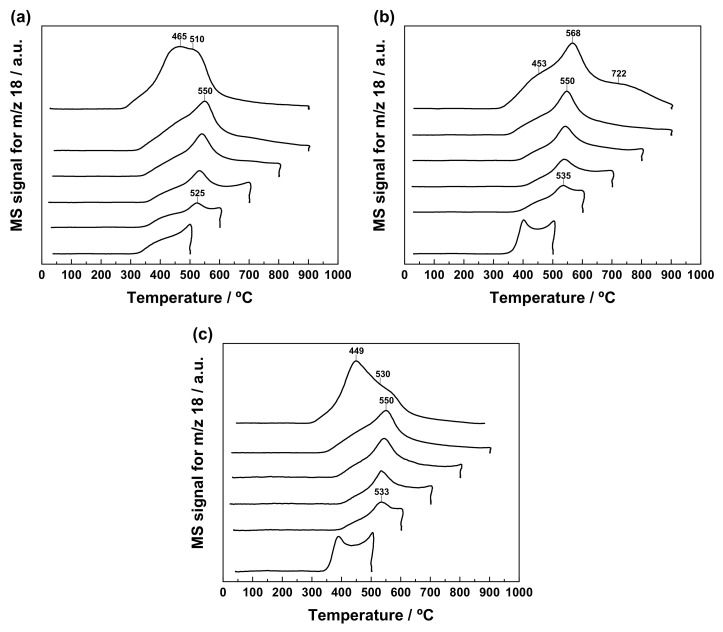
Temperature-programmed reduction (TPR) profiles registered for the undoped and Ln-doped ZrO_2_-CeO_2_ samples during the application of the successive reduction treatments, at increasing temperatures from 500 to 900 °C: (**a**) ZrO_2_-CeO_2_, (**b**) ZrO_2_-Ce_0.87_Y_0.13_O_1.935_, and (**c**) ZrO_2_-Ce_0.87_La_0.13_O_1.935_.

In order to get a more comprehensive understanding of the changes in reducibility taking place as a result of the application of the sequence of thermochemical ageing treatments, the MS signals were recorded for the fresh undoped and Ln-doped nanostructured catalysts during the reduction steps, at increasing temperatures from 500 to 900 °C. Thus, Figure 10 collects the whole set of TPR profiles corresponding to these consecutive reduction treatments, performed on all three nanocatalyst samples. Before analysing these plots, it is worth recalling that each curve in the above figure provides valuable information pertaining to the reducibility of the sample thermally aged at the immediately lower reduction temperatures. As an illustrative example, the TPR diagram registered for the ZrO_2_-CeO_2_ nanocatalyst up to a maximum temperature of 700 °C evidences the redox behaviour of this sample previously submitted to successive reduction treatments at 500 and 600 °C for 1 h under H_2_ (5%)/Ar atmosphere. Furthermore, the thermally aged samples resulting from this series of TPR experiments at increasing temperatures were also subjected to an additional one up to 900 °C, with a view not only to unveil the effects of the application of the consecutive thermochemical ageing treatments on the redox properties of the nanostructured catalysts, but also to compare them with those TPR profiles depicted in Figure 6.

Concerning the reference ZrO_2_-CeO_2_ nanocatalyst, the most relevant changes affecting its reducibility as the maximum temperature of the successive reduction treatments gradually increases are briefly summarized below. First, the major H_2_O evolution peak, which has been previously ascribed to the surface reduction of the supported CeO_2_ nanoclusters, undergoes a slight shift to the right (i.e., from around 525 to 550 °C), accompanied as well by a significant intensity increase, with rising reduction temperature. Such an overall effect may be tentatively associated with two phenomena occurring at least partially over the same temperature range: the sintering of the CeO_2_ crystallites, which displaces the position of the surface reduction peak to higher temperatures, and the plausible formation of cerium-zirconium mixed oxide surface phases, which are well-known to improve the reducibility of pure CeO_2_. Second, the consecutive reduction treatments at increasing temperatures also appear to bring about an enhancement in the relative intensity of the greatly sloped shoulder in respect of the main reduction peak, whereas its onset is apparently shifted by around 30 °C to lower temperatures. Third, the complete removal of the residual carbonate species originally found in the fresh nanocatalyst seems to be fully accomplished after the reduction treatment at 700 °C for 1 h. In fact, the very broad band, peaking at roughly 640 °C in Figure 5, which was connected with the reduction of these species by H_2_, has been completely erased from the trace corresponding to the TPR experiment conducted up to 800 °C. However, it is worth mentioning that the most prominent changes in the redox response are by far observed in the TPR diagram of the thermally aged sample, resulting from the entire series of successive reduction treatments from 500 to 900 °C (sample ZrO_2_-CeO_2_-900, curve at the top of Figure 10a). According to this profile, the application of the thermochemical ageing treatments leads to a material with a markedly improved reducibility. Thus, the reduction of the nanocatalyst starts to be noticeable at temperatures slightly above 250 °C, with H_2_O being mostly evolved in the range from 300 to 600 °C, as a very broad and quite intense band peaking at ca. 465 and 510 °C. Again, such features are compatible with the formation of cerium-zirconium mixed oxide surface phases as a consequence of the consecutive reduction treatments, and especially of that performed at 900 °C. In this connection, from the above TPR results, it becomes evident that this latter reduction temperature marks a breakpoint above which the thermally activated diffusion processes, and their inherent nanostructural changes yielding the aforesaid mixed oxide phases with improved reducibility, take place at a greater rate, and to a much larger extent. This conclusion is strongly in agreement with the results provided by XRD and by XPS, as will be further discussed in greater detail in a later section.

Even more interesting is the comparison with the TPR diagram obtained on the thermally aged ZrO_2_-CeO_2_ sample resulting from a single reduction treatment at 900 °C for 1 h, which was previously shown in Figure 6. Note how this H_2_O evolution profile resembles quite closely that registered for the same reference nanocatalyst formulation after applying the whole set of consecutive reduction treatments from 500 to 900 °C (cf. curve at the top of Figure 10a). Both TPR traces reproduce, to a large extent, the same redox behaviour, with a very broad band spanning over the temperature window from around 275 up to over 600 °C and featuring two poorly defined peaks in the 460 to 550 °C narrow range, as well as a small and badly visible shoulder at 360–370 °C. Nonetheless, the only noticeable differences refer to the temperatures at which these peaks appear, on the one hand, and to their relative intensities, on the other. Regarding the former difference, when submitting to the successive reduction treatments, a slight shift by 20–30 °C towards lower temperatures is observed for both peaks, which now appear at about 465 and 510 °C. In connection with the latter difference, whereas they display essentially the same intensity in the TPR curve for the ZrO_2_-CeO_2_ system previously reduced at 900 °C for 1 h, the application of the series of thermochemical ageing treatments at increasing reduction temperatures leads to a TPR profile, showing a slight, but significant, development of the low temperature peak, at the expense of that centred at a higher temperature.

As far as the evolution of reducibility for the Ln-doped ZrO_2_-CeO_2_ catalytic systems with the consecutive reduction treatments is concerned, from Figure 10b,c, it becomes clear that, irrespective of the dopant, the recorded TPR profiles follow a fairly similar variation pattern to that discussed above for the reference undoped material, up to the reduction treatment at 800 °C. However, the largest differences in the redox response between the undoped and Ln-doped nanocatalysts are found after submitting them to the set of reduction treatments from 500 to 900 °C (i.e., samples ZrO_2_-CeO_2_-900, ZrO_2_-Ce_0.87_Y_0.13_O_1.935_-900, and ZrO_2_-Ce_0.87_La_0.13_O_1.935_-900, whose TPR traces are plotted at the top of Figure 10a–c, respectively). Although their TPR diagrams are all dominated by a very wide band, with H_2_O evolution completing mostly within a window at intermediate temperature values from 300 up to over 600 °C, there also exist some noticeable variations in the relative intensity, and to a lesser extent in the position, of the most intense signals (i.e., peaks and shoulders) that would be worth mentioning. Thus, while the profile for the ZrO_2_-CeO_2_-900 sample exhibits, as its most prominent features, two badly defined peaks located at around 465 and 510 °C, with the former being slightly more intense than the latter (see Figure 10a), the curves recorded for its Ln-doped counterparts display a readily visible and rather intense peak, which is preceded or followed by a shoulder, depending on the dopant. In the case of the Y-containing nanocatalyst, the major peak appears at ca. 568 °C, around 60 °C above the second peak observed in the trace for the ZrO_2_-CeO_2_-900 reference sample, with the shoulder located at about 450 °C. By contrast, for the La-modified system, there is a shift by 25 °C towards lower temperatures of the most intense peak, with respect to its position in the TPR diagram of the undoped nanocatalyst, being followed by the shoulder at higher temperatures (i.e., roughly at 530 °C). All these reducibility changes may be likely ascribed to differences, not only in the extent of cerium integration into the nanoparticulated ZrO_2_ support to form mixed oxide surface phases, but also in the composition of the latter ones, as well as to the presence of the non-reducible Ln^3+^ dopants.

Similarly to the reference ZrO_2_-CeO_2_ material, another important comparison is that established for each of the Ln-doped systems between the ZrO_2_-Ce_0.87_Ln_0.13_O_1.935_-900 sample and that subjected to a single reduction treatment at 900 °C for 1 h (see Figure 6). By comparing their corresponding TPR diagrams, it is concluded that the consecutive thermochemical ageing treatments at increasing reduction temperatures seem to exacerbate the slight differences in the redox response previously pointed out for the samples thermally aged at 900 °C. Thus, for the La-doped nanocatalyst, the most intense peak appearing at roughly 445 °C in the TPR trace obtained on the sample reduced at 900 °C is notably more developed after the successive reduction treatments from 500 to 900 °C. A similar trend is also noticed for the Y-modified material, regarding the evolution of the peak located in the 550–570 °C temperature range.

In summary, the whole set of TPR results commented on up to now suggests that the incorporation of non-reducible Ln^3+^ dopants in the supported CeO_2_ nanoclusters does not notably alter the reduction treatment temperature, at which the most relevant nanostructural modifications resulting in the formation of mixed oxide surface phases, and thereby in the inherent reducibility changes, largely occur. In other words, a reduction treatment at a minimum temperature of 900 °C should be applied, in order to achieve a significant diffusion of cerium species into the bulk of ZrO_2_ crystallites, regardless of whether the supported CeO_2_ phase is doped or not. This conclusion will be further corroborated in the following subsection by means of a systematic XPS study. Furthermore, taking as reference the TPR diagram registered for the ZrO_2_-CeO_2_-900 sample, it becomes apparent that the incorporation of La^3+^ in the surface CeO_2_ phase, followed by the application of the successive thermochemical ageing treatments, leads to a material with improved reducibility at low temperatures, whereas precisely the opposite applies when Y^3+^ is added to the nanocatalyst formulation. Such behaviour is likely to be connected with the extent of the diffusion of cerium and the Ln^3+^ dopant into the bulk of the ZrO_2_ crystallites to yield cerium-zirconium mixed oxide surface phases with enhanced reducibility, as will be discussed in detail below.

#### 3.2.4. Surface Chemical Characterization

Aiming to gain a more detailed insight into the evolution and extent of the integration process between the ZrO_2_ support and the CeO_2_-based surface phase, XPS analyses were carried out on the undoped and Ln-doped ZrO_2_-CeO_2_ nanocatalysts, after applying the consecutive thermochemical ageing treatments at increasing reduction temperatures. Specifically, these experiments allowed one to follow the compositional changes at the outermost surface layers of the samples, where the diffusion processes leading to the formation of the different cerium-zirconium mixed oxide phases are supposed to occur, as a consequence of the aforesaid treatments. In particular, special attention is paid to the variation of the average concentration of both cerium and the Ln dopants in these atomic layers. At this point, it pertains to recall that in the present study the Zr 3*d*, Ce 4*d*, Y 3*d*, and La 4*d* core levels have been considered for quantification purposes, chiefly because these signals come from comparable depths (i.e., around 6.3 nm, which corresponds to three times the IMPF for all these photoelectrons [53]). Furthermore, the (Ce 4*d* + Ln X*d*)/Zr 3*d* ratio has been taken herein as a rough estimate of the integration degree of cerium and the Ln dopant into the ZrO_2_ support for the different thermally aged samples, provided that the (Ce + Ln)/Zr atomic ratios derived from XRF measurements for the freshly prepared nanocatalysts (cf. Table 2) are regarded as the nominal compositions corresponding to a complete integration or mixing of both oxide components to yield homogeneous solid solutions.

**Figure 11:**
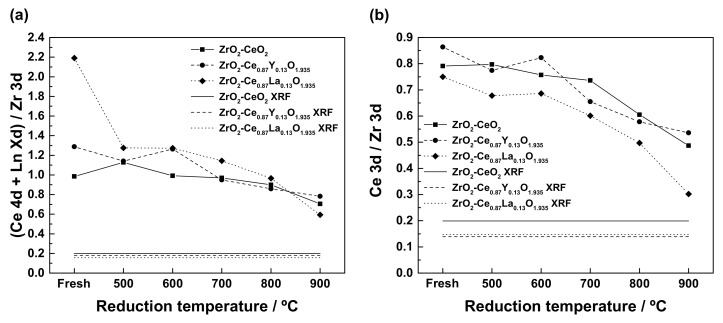
Evolution of the (**a**) (Ce 4*d* + Ln X*d*)/Zr 3*d* and (**b**) Ce 3*d*/Zr 3*d* ratios, with the maximum temperature of the last reduction treatment for the undoped and Ln-doped ZrO_2_-CeO_2_ samples during the application of the successive thermochemical ageing treatments.

The evolution of the (Ce 4*d* + Ln X*d*)/Zr 3*d* ratio for the undoped and Ln-doped ZrO_2_-CeO_2_ nanocatalysts with the maximum temperature of the last applied reduction treatment is displayed in Figure 11a. In this plot, the horizontal straight lines represent the (Ce + Ln)/Zr atomic ratios, as estimated from XRF, for the above-mentioned hypothetical homogeneous solid solutions. From this figure, it becomes clear that all three nanostructured catalyst samples follow a rather similar trend as far as the variation of the (Ce 4*d* + Ln X*d*)/Zr 3*d* ratio with the temperature of the reduction treatment is concerned, irrespective of the nature of the Ln^3+^ dopant. Briefly, this ratio, as a rule, decreases with increasing reduction temperature, thus suggesting a certain diffusion of both cerium and the rare earth dopant into the bulk of the monoclinic ZrO_2_ nanoparticles and the consequent transition from a surface mostly consisting on a cerium-enriched phase to a cerium-zirconium mixed oxide phase. Nevertheless, according to the textural and structural evolution data gathered in Figure 8 and Figure 9, the drop in the (Ce 4*d* + Ln X*d*)/Zr 3*d* ratios for the samples thermally aged up to a reduction temperature of 700 °C may be largely attributed to sintering effects, which entail the growth of the surface CeO_2_-nanoclusters, rather than to the formation of the mixed oxide phases. Moreover, it is also found that these ratios estimated for the materials submitted to the consecutive reduction treatments up to 900 °C are markedly greater as compared to the corresponding (Ce + Ln)/Zr atomic ratios provided by the XRF technique. This fact clearly evidences that cerium and the dopants diffused in a limited extent, so that the cerium-zirconium mixed oxide phases were only formed to a restricted depth into the ZrO_2_ nanoparticle volume.

On the other hand, there also exist some notable differences in the diffusion or integration process between the undoped and Ln-doped nanocatalysts, and between these latter as well, which are worth highlighting. First, in the case of the ZrO_2_-CeO_2_ reference material, the diffusion of cerium inside the ZrO_2_ crystallites seems to be very low, even after the reduction at 800 °C, the decrease in the Ce 4*d*/Zr 3*d* ratio of around 9% for the ZrO_2_-CeO_2_-800 sample with respect to the value calculated for the fresh specimen being mostly ascribable to the sintering of the supported CeO_2_ nanoclusters. Therefore, a minimum reduction temperature of 900 °C is required to attain a significant cerium integration degree, well in agreement with the XRD and TPR results thoroughly discussed in previous sections. Thus, a Ce 4*d*/Zr 3*d* ratio of 0.70 was estimated for the ZrO_2_-CeO_2_-900 sample, which means a drop of about 28.5% in comparison to the freshly prepared nanocatalyst. Note that such a decrease is reasonably comparable to that observed for the undoped material submitted to a single reduction treatment at 900 °C for 1 h (see Table 2). This observation can be regarded as an indication that both this latter and the consecutive reduction treatments at increasing temperatures up to 900 °C result in the diffusion of cerium to a similar extent into the ZrO_2_ nanoparticle volume, thus leading to thermally aged materials featuring almost the same redox response.

Concerning the Ln-doped ZrO_2_-CeO_2_ nanocatalysts, Figure 11a also confirms that, as expected, the incorporation of both Ln^3+^ dopants into the fluorite-type lattice of the CeO_2_-based surface phase significantly influences the diffusion of cerium. By focusing exclusively on this latter process, it should be borne in mind that the above plot is probably not the most suitable one, with a view to following its evolution with the reduction treatment temperature. The reason relies on the fact that the (Ce 4*d* + Ln X*d*)/Zr 3*d* ratio contains an important contribution originating from the rare earth dopants, which makes it difficult to establish proper comparisons between the undoped and Ln-doped catalytic systems with regard to the cerium integration. Aimed at overcoming this drawback, the evolution of the Ce 3*d*/Zr 3*d* ratio, which provides information about the cerium concentration relative to that of zirconium, with the temperature of the last applied reduction treatment, has been depicted in Figure 11b. From this plot, it becomes evident that the extent to which cerium diffuses into the bulk of the ZrO_2_ crystallites is noticeably affected by the presence of both Ln^3+^ dopants. Thus, for the samples ZrO_2_-Ce_0.87_Y_0.13_O_1.935_-900 and ZrO_2_-Ce_0.87_La_0.13_O_1.935_-900, which have been subjected to the whole series of thermochemical ageing treatments, the Ce 3*d*/Zr 3*d* ratio undergoes a decrease of around 38 and 60%, respectively, whereas this drop reaches a 43% for their undoped counterpart. Therefore, from these XPS analyses, it may be concluded that the incorporation of Y^3+^, smaller than both Ce^3+^ and Ce^4+^ cations, into the supported CeO_2_ nanoclusters hinders, to some extent, the diffusion process of cerium into the volume of the ZrO_2_ nanoparticles, and thereby the maximum depth to which the corresponding cerium-zirconium mixed oxide phases are formed. According to previous works, this phenomenon could be connected with the hindering of oxygen mobility in the fluorite lattice, as a consequence of the ordering of the surface oxygen vacancies, which are created during the different reduction treatments, in the vicinity of the Y^3+^ cations [76]. In stark contrast, the opposite effect is observed when the larger La^3+^ dopant is added to the nanocatalyst formulation, thus favouring the diffusion of cerium during the successive reduction treatments.

## 4. Conclusions

By applying a facile surfactant-free controlled chemical precipitation method, a series of three nanostructured catalysts with low total REEs content (i.e., 15 mol.%) was prepared by depositing either CeO_2_ or Ln^3+^-doped CeO_2_ (Ln^3+^ = Y^3+^ or La^3+^; Ln/Ce = 0.15) on the surface of highly crystalline monoclinic ZrO_2_ nanoparticles, as nanometer-sized, fluorite-type clusters. These fresh nanocatalysts were characterized and further subjected to a thermochemical ageing routine, consisting of successive reductions in H_2_ (5%)/Ar at increasing temperatures from 500 to 900 °C, alternated with mild re-oxidations at 350 °C in O_2_ (5%)/He. A detailed study by a surface sensitive technique like XPS, in combination with other chemical characterization techniques, was performed in order to shed light on the diffusion process of cerium into the bulk of the ZrO_2_ support upon reduction to yield Ce_x_Zr_1−x_O_2−δ_ surface phases, featuring enhanced redox properties, as compared to bulk ceria-zirconia mixed oxides and pure ceria. Furthermore, the influence on the aforesaid process of the incorporation in the CeO_2_ lattice of non-reducible trivalent REE cations with size smaller (Y^3+^) and larger (La^3+^) than both Ce^4+^ and Ce^3+^ was also investigated in the present work. The obtained results allow one to draw the following main conclusions:

1. Irrespective of whether the supported CeO_2_ phase is doped or not, and of the nature of the Ln3+ dopant, a reduction treatment at a minimum temperature of 900 °C was required to accomplish a significant diffusion of cerium inside the ZrO_2_ crystallites, with the inherent formation of cerium-zirconium mixed oxide surface phases.
2. The size of the dopant was found to noticeably affect the extent of the diffusion process. As compared to the undoped ZrO_2_-CeO_2_ nanostructured catalyst, Y^3+^ incorporation slightly hindered the cerium diffusion, thereby decreasing the maximum depth to which the corresponding mixed oxide phases are formed, while the opposite effect was observed for the La^3+^-doped nanocatalyst.
3. Such differences in cerium diffusion led to changes in both surface and nanostructural features of the oxides, which were tentatively correlated with the redox response of the thermally aged materials. Thus, the enhanced reducibility in the low temperature range exhibited by the La^3+^-modified nanocatalyst after applying the whole set of consecutive reduction treatments was associated with a greater formation of mixed oxide surface phases. In stark contrast, the slight worsening in reducibility found for the Y^3+^-containing aged sample was interpreted on the basis of a lower amount of the abovementioned surface phases.

## Figures and Tables

**Table 1:** Average macroscopic chemical composition for the fresh undoped and Ln-doped ZrO_2_-CeO_2_ samples.

Sample	ZrO_2_/mol%	CeO_2_/mol%	Ln_2_O_3_/mol%	Ce_0.87_Ln_0.13_O_1.935_/mol%	Ln/Ce/at.%
ZrO_2_-CeO_2_	83.4	16.6	-	-	-
ZrO_2_-Ce_0.87_Y_0.13_O_1.935_	85.8	12.9	1.3	14.2	14.2
ZrO_2_-Ce_0.87_La_0.13_O_1.935_	86.5	11.8	1.8	13.5	14.6

**Table 2:** XPS quantitative results for the fresh undoped and Ln-doped ZrO_2_-CeO_2_ samples.

Sample	(Ce 4*d* + Ln X*d*)/Zr 3*d*	(Ce + Ln)/Zr ^1^/at.%
ZrO_2_-CeO_2_	0.99	0.20
ZrO_2_-Ce_0.87_Y_0.13_O_1.935_	1.29	0.18
ZrO_2_-Ce_0.87_La_0.13_O_1.935_	2.19	0.16

^1^ As determined by XRF.

**Table 3:** Textural parameters estimated for the commercial ZrO_2_ nanoparticles and the fresh undoped and Ln-doped ZrO_2_-CeO_2_ samples.

Sample	*S*_BET_/m^2^·g^−1^	*V*_p_/cm^3^·g^−1^	*D*_p_/nm
ZrO_2_ nanoparticles	35	-	-
ZrO_2_-CeO_2_	84	0.31	3
ZrO_2_-Ce_0.87_Y_0.13_O_1.935_	76	0.35	4
ZrO_2_-Ce_0.87_La_0.13_O_1.935_	63	0.17	3

**Table 4:** XPS quantitative results for the undoped and Ln-doped ZrO_2_-CeO_2_ samples, subjected to a reduction treatment in H_2_(5%)/Ar at 900 °C for 1 h.

Sample	(Ce 4*d* + Ln X*d*)/Zr 3*d*	(Ce + Ln)/Zr ^1^/at.%
ZrO_2_-CeO_2_	0.65	0.20
ZrO_2_-Ce_0.87_Y_0.13_O_1.935_	0.67	0.18
ZrO_2_-Ce_0.87_La_0.13_O_1.935_	0.73	0.16

^1^ As determined by XRF.

## Supplementary Materials

The following are available online at https://www.mdpi.com/1996-1944/13/12/2818/s1, Figure S1: (**a**) Evolution with temperature of mass/charge (*m*/*z*) ratios 18 and 28 during the TPR-MS analysis of the commercial ZrO_2_ nanoparticles used as support, and (**b**) comparison of water evolution curves for the bare nanoparticles and the fresh ZrO_2_-CeO_2_ sample, Figure S2: Evolution with temperature of mass/charge (*m*/*z*) ratios 2 and 28 during the TPR-MS analysis of the fresh samples: (**a**) ZrO_2_-CeO_2_, (**b**) ZrO_2_-Ce_0.87_Y_0.13_O_1.935_, and (**c**) ZrO_2_-Ce_0.87_La_0.13_O_1.935_, Figure S3: Evolution with temperature of mass/charge (*m*/*z*) ratio 30 during the TPR-MS analysis of the fresh samples: (**a**) ZrO_2_-CeO_2_, (**b**) ZrO_2_-Ce_0.87_Y_0.13_O_1.935_, and (**c**) ZrO_2_-Ce_0.87_La_0.13_O_1.935_.
